# Effects of evening smartphone use on sleep and declarative memory consolidation in male adolescents and young adults

**DOI:** 10.1093/braincomms/fcae173

**Published:** 2024-05-17

**Authors:** Christopher Höhn, Michael A Hahn, Georg Gruber, Belinda Pletzer, Christian Cajochen, Kerstin Hoedlmoser

**Affiliations:** Laboratory for Sleep, Cognition and Consciousness Research, Department of Psychology, Paris Lodron University of Salzburg, 5020 Salzburg, Austria; Centre for Cognitive Neuroscience Salzburg (CCNS), Paris Lodron University of Salzburg, 5020 Salzburg, Austria; Hertie-Institute for Clinical Brain Research, University Medical Center Tübingen, 72076 Tübingen, Germany; The Siesta Group Schlafanalyse GmbH, 1210 Vienna, Austria; Centre for Cognitive Neuroscience Salzburg (CCNS), Paris Lodron University of Salzburg, 5020 Salzburg, Austria; Centre for Chronobiology, Psychiatric Hospital of the University of Basel, 4002 Basel, Switzerland; Research Cluster Molecular and Cognitive Neuroscience (MCN), University of Basel, 4055 Basel, Switzerland; Laboratory for Sleep, Cognition and Consciousness Research, Department of Psychology, Paris Lodron University of Salzburg, 5020 Salzburg, Austria; Centre for Cognitive Neuroscience Salzburg (CCNS), Paris Lodron University of Salzburg, 5020 Salzburg, Austria

**Keywords:** smartphone, short-wavelength light, sleep, melatonin, memory consolidation

## Abstract

Exposure to short-wavelength light before bedtime is known to disrupt nocturnal melatonin secretion and can impair subsequent sleep. However, while it has been demonstrated that older adults are less affected by short-wavelength light, there is limited research exploring differences between adolescents and young adults. Furthermore, it remains unclear whether the effects of evening short-wavelength light on sleep architecture extend to sleep-related processes, such as declarative memory consolidation. Here, we recorded polysomnography from 33 male adolescents (15.42 ± 0.97 years) and 35 male young adults (21.51 ± 2.06 years) in a within-subject design during three different nights to investigate the impact of reading for 90 min either on a smartphone with or without a blue-light filter or from a printed book. We measured subjective sleepiness, melatonin secretion, sleep physiology and sleep-dependent memory consolidation. While subjective sleepiness remained unaffected, we observed a significant melatonin attenuation effect in both age groups immediately after reading on the smartphone without a blue-light filter. Interestingly, adolescents fully recovered from the melatonin attenuation in the following 50 min before bedtime, whereas adults still, at bedtime, exhibited significantly reduced melatonin levels. Sleep-dependent memory consolidation and the coupling between sleep spindles and slow oscillations were not affected by short-wavelength light in both age groups. Nevertheless, adults showed a reduction in N3 sleep during the first night quarter. In summary, avoiding smartphone use in the last hour before bedtime is advisable for adolescents and young adults to prevent sleep disturbances. Our research empirically supports general sleep hygiene advice and can inform future recommendations regarding the use of smartphones and other screen-based devices before bedtime.

## Teaser

Höhn *et al*. show that nighttime smartphone use suppresses melatonin secretion significantly longer in adults than in adolescents who can recover if the smartphone is switched off 50 min before bedtime. These results support recommendations to avoid LED screens in the hour before bedtime to protect sleep quality and physiology.

## Graphical Abstract

**Graphical Abstract:**
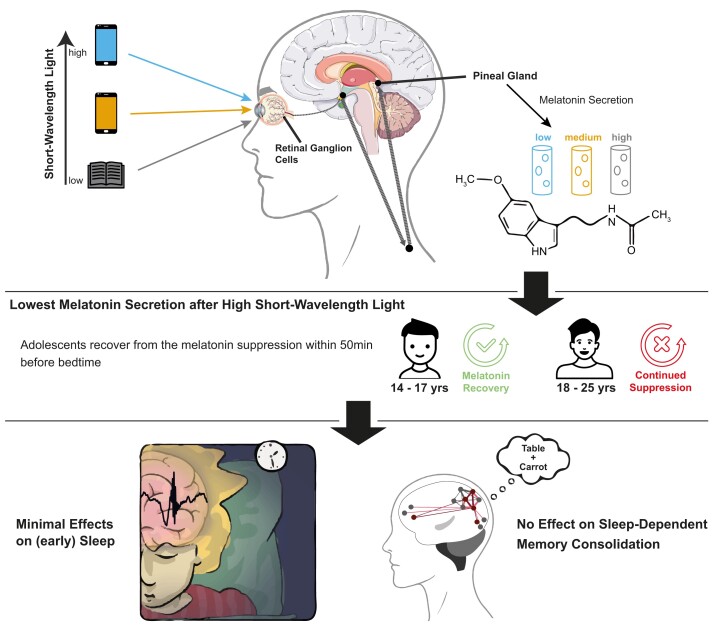

## Introduction

Evening exposure to artificial short-wavelength light has greatly increased over the last decades due to the widespread use of portable devices with LED screens.^1,2^ Consequently, the potentially adverse effects on subsequent sleep have received considerable attention, although it remains unclear whether and under what circumstances specific sleep-related processes, such as sleep-dependent memory consolidation, are affected.

Commonly, LED screens have a spectral distribution peaking within 450–500 nm and are highly effective in attenuating nocturnal melatonin secretion^3-7^ and sleepiness.^8-10^ These light-induced effects are triggered by the stimulation of intrinsically photosensitive retinal ganglion cells (ipRGCs)^11,12^ most sensitive to short-wavelength light around 480 nm,^13^ which is close to the spectral peak of LED screens. Short-wavelength light stimulating the melanopsin-containing ipRGCs entrains circadian rhythms via the suprachiasmatic nuclei (SCN) in the hypothalamus.^14,15^ Consequently, the short-wavelength light-dependent ipRGC stimulation leads to an activation of melanopsin, which suppresses the secretion of melatonin from the pineal gland and induces a successive alerting response via different neuronal connections of the SCN.^15-17^ While this mechanism is crucial for synchronizing the human physiology with the natural light-and-dark cycle, artificial lighting can disrupt that process by simulating daytime when natural light is absent.^18^ Such a disruption can delay bedtimes or sleep onset in general,^8,9,19,20^ increase sleep fragmentation,^21-23^ reduce or delay EEG slow-wave activity (<4.5 Hz)^24-26^ and can even diminish N3 sleep,^27-29^ during which the intricate interplay between slow oscillations (SOs) and sleep spindles governs sleep-dependent memory consolidation.^30-33^ Additionally, evening light exposure has been reported to elicit longer-lasting effects on next-morning cognitive performance^8,34^ and mood.^35^ Therefore, specific sleep-related functions that are necessary for behavioural performance, such as memory consolidation, might also be disturbed by evening light exposure. However, research assessing potential light-induced effects on sleep-dependent memory consolidation and its electrophysiological markers during sleep is not yet available.

To mitigate disruptive effects of evening light exposure, different ‘blue-light-blocking’ strategies have emerged, which reduce the emitted short-wavelength light by either changing screen characteristics or by wearing of specialized glasses. These filters have been shown to reduce the impact on alertness and subsequent sleep in some studies.^36-39^ However, there is also a body of research that has failed to demonstrate significant light-induced sleep effects altogether,^40-43^ suggesting that the reported light effects on sleep might be dependent on specific aspects of the study designs and light sources, such as the melanopic equivalent daylight illuminance (m-EDI).^44^

Furthermore, recent evidence indicates that the impact of light on circadian rhythms—and especially on sleep—varies significantly across individuals,^45-48^ with distinct age groups experiencing disparate effects.^49-51^ Since the strongest age effects are attributed to lens yellowing in older individuals,^52^ the majority of research has focussed on comparing older and younger adults (>50 years versus 18–30 years).^53,54^ Studies comparing light sensitivity between age groups transitioning from adolescence to adulthood are scarce.^53^ This is surprising, since adolescents, especially those in early-to-mid puberty, have been held up as the most sensitive to evening light exposure in terms of acute melatonin suppression^55,56^ and are known to often use their smartphones until shortly before bedtime. Therefore, it remains unclear whether there are developmental differences in light sensitivity between late adolescents and young adults and whether they differ regarding longer-lasting effects of evening light exposure.

Here, we implemented an extensive within-subject study design to assess how evening smartphone use (with or without a blue-light filter) affects not only circadian processes and sleep in general, but also sleep-dependent memory consolidation. We further address the question of whether potential light-induced effects differ between adolescents (14–17 years) and young adults (18–25 years).

## Materials and methods

### Participants

Data were recorded from 68 male subjects, including 33 adolescents (14–17 years, mean: 15.42 ± 0.97) and 35 young adults (18–25 years, mean: 21.51 ± 2.06). We recorded at least 30 participants without missing data per age group to be able to reasonably expect normally distributed outcomes. Recording significantly more participants would not have been feasible due to the extensive nature of the study design, which imposed time and funding constraints. All participants were recruited by online advertisements or by visiting schools and university lectures in Salzburg. Some participants were excluded from certain analyses because of technical issues, missing data, or poor data quality. The sample size for each analysis varied within *N* = 61–66 and is provided in the respective paragraph or figure caption.

We focussed on male participants to avoid sex differences in sleep^57,58^ and light sensitivity^47^ and because comparing males and females within a single age group would have been challenging due to the asynchronous onset of puberty. All participants were non-smokers, free of medication and identified as neutral, mild-evening or mild-morning types.^59^ Further inclusion criteria were right-handedness, no drug abuse, no shiftwork and a regular sleep rhythm (8 h per night at consistent bedtimes with a variation of ±30 min), which was monitored by wrist actigraphy (MotionWatch 8; CamNtech Ltd., Cambridge, England) and daily sleep logs (see Supplementary Table 1 for an overview of all screening questionnaire results).

Adults received either 100€ and 16 h of university course credit or 50€ and 24 h of course credit. Adolescents were remunerated with 200€. The study was approved by the local ethics committee (ethics agreement: GZ16/2014—addendum 2020) and was conducted in accordance with the guidelines of the Declaration of Helsinki.^57^

### Study design

**Figure 1:**
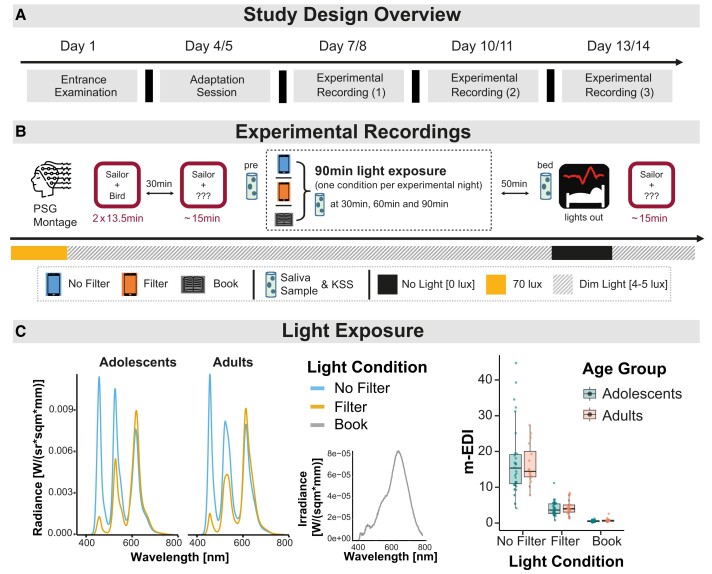
**Methodological overview**. (**A**) Outline of the 14-day study protocol. (**B**) Detailed procedure of the three experimental recordings. Each recording started 5 h before the participants’ habitual bedtime. After the PSG setup, the encoding sessions of the declarative memory task were performed, followed by a first immediate recall session after ∼30 min. After the recall session, participants provided a saliva sample and rated their subjective sleepiness on the Karolinska Sleepiness Scale (‘pre’) before engaging in a 90 min reading session, either on a smartphone without (No Filter) or with (Filter) a blue-light filter, or via a printed book (Book). Short breaks of maximum 5 min were scheduled after every 25 min of reading for additional saliva samples and sleepiness ratings (c.f., the ‘30 min’, ‘60 min’ and ‘90 min’ marks). The order of the light conditions was randomized for each subject. Bedtime was scheduled ∼50 min after the end of the reading session, and a final saliva sample and sleepiness rating were obtained (‘bed’). The next morning, following an 8 h sleep opportunity, the delayed recall session was conducted. Room lights were set at 4–5 lx throughout the recording, except for the PSG montage (70 lx) and the 8 h sleep window (0 lx). (**C**) Light spectra obtained by spectrometry (left side) and light intensities (m-EDI) recorded at eye level during the 90 min light exposure for each subject (right side, *N*_Adolescents_ = 32; *N*_Adults_ = 20, individual data points represent single subject values). Although adolescents and adults used different smartphones (Samsung Galaxy A51 versus Samsung Galaxy A50), the light spectra and m-EDI were highly comparable between the age groups [main effect of age group in a mixed-design ANOVA: *F*(1,36) = 0.02; *P* = 0.885]. As anticipated, m-EDI was highest in the No Filter condition and lowest in the Book condition [main effect of condition: *F*(1.05,37.91) = 109.77; *P* < 0.001; no significant interaction between age group and condition: *F*(1.05,37.91) = 0.20; *P* = 0.674].

Each participant engaged in the study for ∼14 days (see Fig. 1A) between October 2019 and August 2022. Inclusion criteria were checked, and informed consent was obtained on the first day at the sleep laboratory of the University of Salzburg. On this occasion, participants also received the actigraph, which was worn for the duration of the study. Furthermore, they started to fill in daily sleep diaries.^58^ On their next visit (Day 4), participants arrived 2 h before their habitual bedtime for an adaptation night to avoid potential first-night effects.^59,60^ On Days 7, 10 and 13, the experimental recordings were scheduled. The experimental paradigm started 5 h before habitual bedtime. After applying electrodes for the polysomnography (PSG) recording under 70 lx room lighting (Emilum GmbH, Oberalm, Austria), lights were dimmed to 4–5 lx, and the participants performed several tasks and filled in questionnaires throughout the evening (a detailed outline of the complete protocol can be found in previous publications).^23,61^ Room lights were kept at 4–5 lx until bedtime, and bathroom breaks were scheduled at standardized time points between tasks. When participants left the laboratory for a bathroom break, they wore blue-light-filtering, orange-tinted glasses (Uvex, Honeywell, Charlotte, NC, USA).

A declarative memory task^62^ was conducted after placing the electrodes for the PSG. The encoding session of the memory task was followed after approximately 30 min by an immediate cued-recall session and then, the next morning, by a delayed recall session. After the immediate recall, the first saliva sample and subjective sleepiness rating were obtained before the light exposure started (‘pre’, see Fig. 1B). Subjective sleepiness was rated on a German adaptation of the Karolinska Sleepiness Scale (KSS),^63^ which is a 10-point Likert scale (instead of the classic 9-point version),^64^ ranging from 1 (‘extremely alert’) to 10 (‘extremely sleepy’).

The light exposure comprised reading standardized stories (see Supplementary Table 2) for 90 min with short breaks of maximum 5 min after every 25 min to obtain a saliva sample and sleepiness rating (‘30 min’, ‘60 min’ and ‘90 min’ marks in Fig. 1B). Participants either read on a smartphone without a blue-light filter (No Filter), with a blue-light filter (Filter) or in a printed book (Book) in each experimental recording. To ensure that the participants were reading, we asked them a few simple questions about the content after each reading block. Approximately 50 min (overall mean: 49.35 min, see Supplementary Table 3) passed between the end of the light exposure and bedtime (average laboratory bedtime for adults was 23:08 ± 00:25 and for adolescents 22:39 ± 00:39). The next morning, following a sleep opportunity of 8 h, participants were woken up and concluded the experimental recording with the delayed recall session of the declarative memory task.

### Light exposure

During the light exposure, background room lighting was kept within 4–5 lx. In the Book condition, this was the only light source, but participants did not report difficulties during reading, and no significant difference in the overall brightness perception or exhaustion was observable among the light conditions (see Supplementary Table 4). In the smartphone conditions (No Filter and Filter), standardized smartphones were used (Samsung Galaxy A50 and A51 enterprise editions; Samsung Electronics, Seoul, Korea), with standardized display brightness. Adults used the A50 smartphone, while adolescents used the A51 (this did not significantly affect the light characteristics—see Fig. 1C). In the Filter condition, the built-in ‘blue-light’ filter was activated to reduce the amount of emitted short-wavelength light. The light spectra of the smartphones and the room lighting spectrum (i.e. the light source in the Book condition) were measured with a spectrometer (see Fig. 1C—left side—and Supplementary Table 5; JETI spectraval 1501; JETI Technische Instrumente GmbH, Jena, Germany). These measurements were conducted at 37 cm to the device and at a height of 88 cm, representing a realistic reading position. During each experimental recording, participants wore special windowless glasses with a sensor (Luxblick 2.0; Ilmenau University of Technology, Ilmenau, Germany) that measured the light intensity at the individual eye level (see Fig. 1C—right side).

### Declarative memory task

During the encoding session of the memory task, 80 unrelated word pairs were presented for 1.5 s each, and a fixation cross was shown for 8.5 s in between each word pair. Two encoding sessions were completed with a different order of the word pairs. A different but similarly difficult set of word pairs was used for each experimental recording (see Supplementary Fig. 1 for a comparison of the different word pair lists). During recall, only the first word of a pair was presented. Participants were asked to press a button on a response time box (RTBox v5/6; Ohio State University, Columbus, USA) when they remembered the missing word and then vocalized the word. The experimenter noted the answer and marked it as ‘correct’, ‘semantically correct’, ‘incorrect’ or ‘missing’. After each button press or when 6.5 s had elapsed, a fixation cross was presented for 3.5 s. We calculated the task performance in the task as the percentage of correctly recalled word pairs relative to all 80 word pairs. We decided not to include semantically correct answers to avoid subjective decisions. Overnight memory consolidation was obtained by subtracting the immediate recall performance from the delayed recall performance the next morning and dividing it by the immediate recall performance (baseline). Multiplied by 100, this expresses the percentage change in overnight memory performance.

### PSG

Eleven gold cup electrodes (Grass Technologies, Astro-Med GmbH, Rodgau, Germany) were applied to the positions F3, Fz, F4, C3, Cz, C4, P3, Pz, P4, O1 and O2, according to the 10–20 system. Fpz was used as ground electrode. Two vertical and two horizontal electrooculography electrodes were mounted, as well as two electromyography electrodes on the musculus mentalis, and two electrodes on the mastoids (A1 and A2). All data were recorded with 500 Hz using a BrainAmp system (Brain Products GmbH, Munich, Germany) and BrainVision Recorder software (v2.11; Brain Products GmbH, Munich, Germany, 2015). Impedances were always kept below 10 kΩ. We used an automatic sleep-staging approach (Somnolyzer 24x7; The Siesta Group GmbH, Vienna, Austria), which was controlled by a human expert scorer, based on the American Academy of Sleep Medicine criteria.^65^

### EEG analyses

Scalp EEG data were scanned automatically for major artefacts in BrainVision Analyzer (v2.2; Brain Products GmbH, Munich, Germany, 2019) with the following artefact criteria: voltage steps exceeding 50 μV/ms, absolute voltage difference of >400 μV within 200 ms or <0.5 μV for >100 ms. The results were visually cross-validated and manually overruled if the detection yielded major false positives or missed large artefacts. Afterwards, the EEG data and bad intervals were exported for further analyses in MATLAB (R2021b; MathWorks Inc., Natick, USA). The data were demeaned and re-referenced against the common average of all scalp channels. All data processing in MATLAB was achieved by custom code that was written using functions from Fieldtrip,^66^ EEGlab^67^ and the CircStat toolbox.^68^

#### Slow-wave activity

EEG spectral power within 0.5–4 Hz was analysed during N3 across the whole night and the first night quarter (i.e. first 2 h of sleep) in artefact-free 5 s epochs. Power spectra were calculated in Fieldtrip^66^ by applying the FOOOF algorithm^69^ to separate the oscillatory component from aperiodic background activity. A Hanning window was chosen, and frequencies from 0 to 20 Hz were analysed with a resolution of 0.2 Hz (Rayleigh frequency). The power values were averaged across the whole night or the first quarter and *Z*-standardized within each subject to represent the deviation in SWA from the subject's average across the experimental recordings.

#### SO–spindle coupling

We followed an established approach to assess the coupling between SOs and sleep spindles in an individualized manner.^30,70^ First, the individual spindle peak frequency during non rapid eye movement sleep was determined for each recording by obtaining the oscillatory component of the EEG power spectra. For these analyses, the epoch length was set to 15 s to obtain a better frequency resolution (0.067 Hz). The individual spindle peak was defined as the highest peak in the oscillatory component of the power spectrum within 10–17 Hz and smoothed over frontal, central and parietal electrodes. Next, individual spindle and SO events were detected automatically for each channel. Given the EEG setup with 11 scalp electrodes, it was not feasible to trace simultaneously detected events across multiple channels back to their topographical origin in source space. Therefore, all detected events were treated separately as in previous studies.^30,71^ While each electrode is considered for topographical analyses, we focussed on Fz for all main analyses.

For spindle detection, data were filtered within ±2 Hz around the individually detected spindle peak frequency before applying a Hilbert transformation and smoothing the signal with a 200 ms moving average. Spindle events were identified by the algorithm when the 75th percentile of the amplitude was exceeded for a duration of 0.5–3 s. SO events were detected by bandpass filtering the data within 0.3–2 Hz, followed by obtaining all zero-crossings of the signal. An SO was identified when the zero-crossings were separated by 0.8–2 s, and the amplitude criterion exceeded the 75th percentile. All SO and spindle events were stored as epochs by spanning ±2.5 s around the amplitude trough of the detected SO (i.e. minimum value of the 0.3–2 Hz filtered signal) or around the amplitude peak of the detected spindle (i.e. maximum value of the bandpass-filtered in the individual spindle frequency range). Epochs containing artefacts were discarded.

We analysed the coupling between SOs and spindles with an event-locked, cross-frequency coupling approach for each channel but focussed mainly on the electrode Fz, in accordance with previous research.^30,71^ After *Z*-transforming each epoch, we extracted the phase angle of the SO component in the 2 Hz low-pass filtered data at the corresponding spindle amplitude peak.^30^ To verify that exclusively epochs in which a spindle occurred within an SO were included, we cross-referenced the epochs and selected only SO trials in which a spindle occurred within ±1.5 s around the SO trough.

Visualization of the coupling results and calculation of the phase angle and coupling strength [mean vector length (mvl), i.e. the degree of phase dependence between two oscillations] was achieved using the CircStat toolbox^68^ and by obtaining event-locked time–frequency representations (TFRs) centred on the SO troughs. These TFRs were calculated by applying a 500 ms Hanning window on the demeaned and detrended SO epoch data and then implementing a time–frequency analysis from 5 to 30 Hz, with a frequency resolution of 0.5 Hz from −2 to 2 s around the SO troughs in steps of 50 ms. The resulting TFR was baseline corrected and *Z*-transformed with a bootstrapped baseline distribution (5000 iterations) from −2 to −1.5 s.^30^

### Melatonin

Participants were instructed not to eat chocolate or bananas on the experimental days and to brush their teeth at least 1 h before the first sample. For each sample, a minimum of 2 ml saliva was obtained using 15 ml saliva tubes (Greiner Bio-One, Kremsmünster, Austria). Samples were stored at −20°C before they were centrifuged twice (15 and 10 min) to isolate mucus and solid particles. After centrifugation, the samples were conserved again at −20°C. Melatonin concentrations in the adult cohort were assessed twice with Salimetrics salivary ELISA kits (Salimetrics LLC, State College, USA) and in the adolescent cohort with Novolytix salivary ELISA kits (NovoLytiX GmbH, Witterswil, Switzerland). A technical comparison between the two melatonin kits can be found in Supplementary Table 6, and all analyses of the melatonin data relied on within-subject changes. Melatonin concentrations below or above the lowest or highest standards were set to the corresponding detection threshold. We analysed the melatonin levels from before the light exposure (‘pre’) and 25–30 min, 55–60 min and 85–90 min after the onset of the light exposure (‘30 min’, ‘60 min’ and ‘90 min’ marks on Fig. 1B). A final sample was analysed directly before bedtime (‘bed’). Missing values were interpolated by calculating the average of a participant's two adjacent samples (this affected only 1.7% of the samples). For further analyses, the melatonin concentration of each sample was baseline corrected to the respective saliva sample before the light exposure. Additionally, we computed the melatonin recovery, which we defined as the increase from immediately after the end of the light exposure until bedtime. A higher melatonin recovery therefore corresponds to a quicker dissipation of the melatonin suppression effect.

### Statistical analyses

The sleepiness data were analysed by means of non-parametrical ANOVA-type procedures^72,73^ to account for the lack of interval-scaled data. For all other data, multi-factorial mixed ANOVAs were computed with the between-subject factor ‘age group’ and the within-subject factors ‘light condition’ and ‘timepoint’. Analyses that included splitting the data into the first night quarter and the whole night were conducted separately. Spearman rank correlations were calculated. When multiple comparisons were performed, the Benjamini–Hochberg^74^  *P*-value correction was used. For statistics on the SO–spindle coupling data, we used the circ_r^68^ function in MATLAB to obtain the average coupling strength (i.e. mvl) and calculated the percentage of spindles locked to the SO up-state (i.e. within ±22.5° around the SO peak) relative to all co-occurring SO–spindle events.^30^ Relative treatment effects (RTEs) are reported for the non-parametrical ANOVA-type statistics. The RTE describes the probability of a randomly chosen value from one condition being larger than a randomly chosen value from the remaining dataset (i.e. all other conditions). All statistical tests were two tailed, and the significance level was set to 0.05 (*P*-values below 0.10 were considered statistical trends). RStudio (v2022.7.2.576; RStudio, Boston, USA) and MATLAB were used for all statistical analyses and visualizations.

## Results

**Figure 2:**
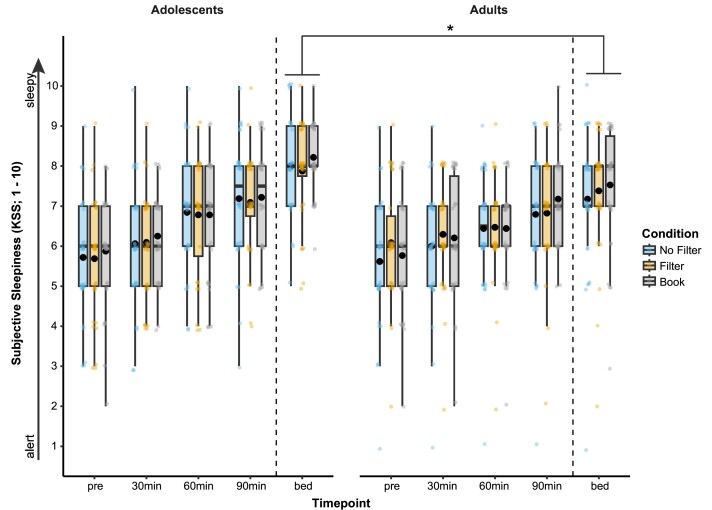
**Subjective sleepiness**. Trajectory of subjective sleepiness ratings on the KSS throughout the evening, starting immediately before the light exposure (‘pre’) and assessed every 30 min throughout the reading session (‘30 min’, ‘60 min’ and ‘90 min’) as well as at bedtime (‘bed’; ca. 50 min after the light exposure). Boxplots are shown together with single datapoints and the corresponding mean sleepiness ratings (bold dots inside the boxplots). At bedtime, the adolescents felt significantly sleepier than the adults (non-parametric *t*-test: *T*_64_ = −2.23; *P* = 0.026), but no effect of light condition emerged (non-parametric ANOVA statistic: *ATS*_6_ = 0.99; *P* = 0.428). The dashed vertical line indicates that there was a break of 50 min between the end of the light exposure (‘90 min’ mark) and bedtime. *P_adj._ ≤ 0.05. *N*_Adolescents_ = 32 and *N*_Adults_ = 34.

The light conditions had no significant effect on the sleepiness ratings over the course of the evening (ATS_6_ = 0.99; *P* = 0.428; see Fig. 2). As expected, subjective sleepiness increased significantly over the evening in all conditions and age groups [ATS_2.54_ = 125.66; *P* < 0.001; RTE_(pre)_ = 0.34, RTE_(30min)_ = 0.40, RTE_(60min)_ = 0.49, RTE_(90min)_ = 0.57, RTE_(bed)_ = 0.70]. However, an interaction between the factors ‘age group’ and ‘timepoint’ emerged (ATS_2.54_ = 4.48; *P* = 0.006), indicating that adolescents felt significantly sleepier at bedtime than adults did [*T*_64_ = −2.23; *P* = 0.026; RTE_(Adolescents:bed)_ = 0.76, RTE_(Adults:bed)_ = 0.65].

**Figure 3:**
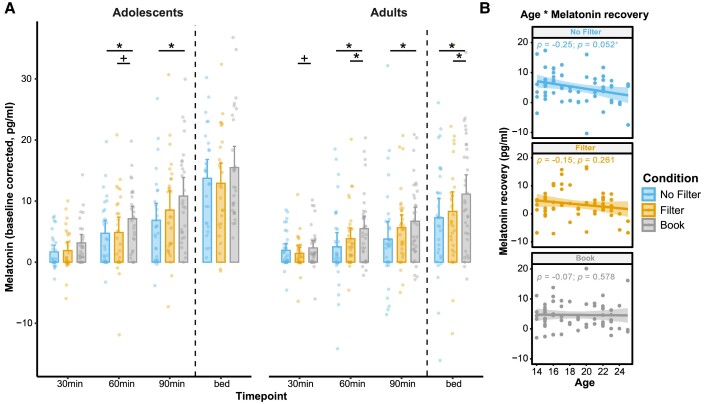
**Melatonin effects**. (**A**) Baseline-corrected melatonin concentration throughout the evening (i.e. relative change of melatonin concentration from ‘pre’ light exposure) assessed with a mixed-design ANOVA. Overall, melatonin increased throughout the evening [*F*(1.63,96.37) = 82.60; *P* < 0.001] and was highest at bedtime (*post hoc* tests; bed versus 90 min: *t*_60_ = 9.04; *P*_adj._ < 0.001, bed versus 60 min: *t*_60_ = 8.26; *P*_adj._ < 0.001, bed versus 30 min: *t*_60_ = 10.40; *P*_adj._ < 0.001) with a greater increase in the adolescent group [*F*(1.70,46.02) = 70.08; *P* < 0.001 versus *F*(1.56, 50.07) = 22.18; *P* < 0.001]. As expected, a clear melatonin attenuation was observable in the No Filter and Filter smartphone conditions during light exposure (*post hoc* tests; Adolescents—60 min, No Filter versus Book: *t*_27_ = −2.68; *P*_adj._ = 0.038 and Filter versus Book: *t*_27_ = −2.21; *P*_adj_. = 0.054, Adolescents—90 min, No Filter versus Book: *t*_27_ = −2.92; *P*_adj_. = 0.021, Adults—30 min, Filter versus Book: *t*_32_ = −2.35; *P*_adj._ = 0.075, Adults—60 min, No Filter versus Book: *t*_32_ = −2.81; *P*_adj._ = 0.013, Filter versus Book: *t*_32_ = −2.97; *P*_adj._ = 0.013, Adults—90 min, No Filter versus Book: *t*_32_ = −2.76; *P*_adj._ = 0.029). For adults only, this effect lasted until bedtime (No Filter versus Book: *t*_32_ = −2.78; *P*_adj._ = 0.027, Filter versus Book: *t*_32_ = −2.36; *P*_adj._ = 0.036). Bars represent the mean and error bars the 95% confidence interval around the mean. (**B**) Spearman rho correlations between participant age and the change in melatonin from immediately after light exposure (90 min) to bedtime (bed). In the smartphone condition without a blue-light filter (No Filter), older subjects showed, by trend, a reduced melatonin recovery. *P_adj._ ≤ 0.05. ^+^*P*_adj._ ≤ 0.1. *N*_Adolescents_ = 28 and *N*_Adults_ = 33.

On a physiological level, the baseline-corrected melatonin secretion over the evening was affected by both light condition [*F*(4.07,240.38) = 2.92; *P* = 0.021; *η*^2^_part._ = 0.05] and age group [*F*(1.63,96.37) = 5.65; *P* = 0.008; *η*^2^_part._ = 0.09; see Fig. 3A]. These effects indicated a stronger increase in salivary melatonin concentration for the adolescents [*F*(1.70,46.02) = 70.08; *P* < 0.001; *η*^2^_part._ = 0.72] than for the adults [*F*(1.56,50.07) = 22.18; *P* < 0.001; *η*^2^_part._ = 0.41]. Furthermore, the interaction between timepoint and light condition indicated a clear melatonin attenuation effect, especially in the No Filter condition after the end of the light exposure for both age groups [*F*(2,118) = 9.17; *P* < 0.001; *η*^2^_part._ = 0.13, see pairwise comparisons in Fig. 3A]. At bedtime (i.e. ∼50 min later), it became evident that the significant attenuation effect had vanished for the adolescents [*F*(2,54) = 1.68; *P* = 0.195; *η*^2^_part._ = 0.06] but was still present for the adults [*F*(2,64) = 5.41; *P* = 0.007; *η*^2^_part._ = 0.15], with significantly lower melatonin concentrations in both smartphone conditions than in the Book condition (No Filter versus Book: *t*_32_ = −2.78; *P*_adj._ = 0.027; *d* = −0.43, Filter versus Book: *t*_32_ = −2.36; *P*_adj._ = 0.036; *d* = −0.32). When the amount of melatonin recovery from the end of light exposure (‘90 min’) to bedtime (‘bed’) is directly compared between adolescents and adults, it becomes evident that the adolescents recovered significantly more melatonin than the adults (*t*_59_ = 2.68; *P* = 0.010; *d* = 0.70). This finding of a quicker melatonin recovery by adolescents can be substantiated by correlating the change in melatonin concentration from the end of the exposure to bedtime with participant age. Here, in the No Filter smartphone condition (i.e. the condition with the strongest melatonin suppression effect), a negative correlation emerged between melatonin recovery and participant age, indicating that older subjects showed a trend of smaller melatonin recovery (*rho*_59_ = −0.25; *P* = 0.052, see Fig. 3B).

**Figure 4:**
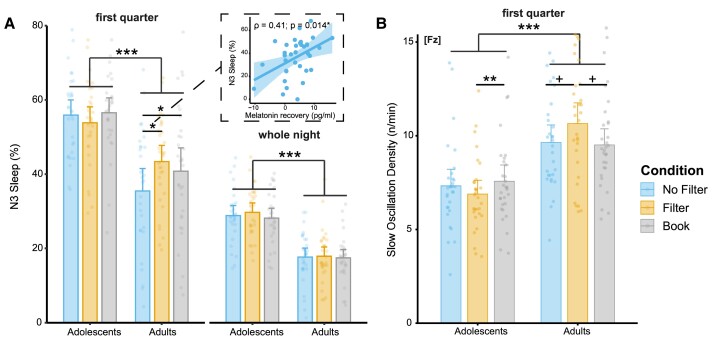
**Sleep architecture and physiology**. (**A**) Overall, adolescents showed a higher percentage of N3 sleep based on mixed-design ANOVAs [first quarter: *F*(1,60) = 29.70; *P* < 0.001, whole night: *F*(1,60) = 45.51; *P* < 0.001], but only the sleep of adults was affected in the smartphone condition without a blue-light filter (No Filter), indicated by less N3 during the first night quarter (*post hoc* tests; No Filter versus Filter: *t*_32_ = −2.90; *P*_adj._ = 0.020, No Filter versus Book: *t*_32_ = −2.38; *P*_adj._ = 0.036). Adults with a higher melatonin recovery before bedtime also had a higher percentage of N3 during the first night quarter. (**B**) During the first night quarter, the density of detected SOs in N3 was significantly different between age groups and light conditions as assessed by a mixed-design ANOVA [interaction: *F*(2,116) = 5.90; *P* = 0.004]. Overall, adults showed a higher SO density than did adolescents [main effect of age group: *F*(1,58) = 25.10; *P* < 0.001]. Adolescents had a significantly lower SO density in the Filter than in the Book condition (*t*_29_ = −3.27; *P*_adj._ = 0.008) and adults, by trend, a higher density in the Filter than in the No Filter (*t*_29_ = 1.96; *P*_adj._ = 0.089) or Book (*t*_29_ = 2.23; *P*_adj._ = 0.089) condition as indicated by *post hoc* tests. ***P_adj._ < 0.001. **P_adj._ ≤ 0.01. *P_adj._ ≤ 0.05. ^+^*P*_adj._ ≤ 0.1. *N*_Adolescents_ = 30 and *N*_Adults_ = 32. Bars display the mean and error bars the 95% confidence interval.

Despite the effects of the smartphone conditions on melatonin secretion, the general sleep architecture remained largely unaffected (see Table 1). Based on previous findings,^25,27,29^ we focussed in more detail on (early) deep sleep and investigated whether the light exposure from the smartphone influenced more subtle measures during N3 sleep. We analysed light effects during the first night quarter and across the whole night, including the percentage of N3, the density of SOs and the SWA (0.5–4 Hz). Across the whole night, the N3 percentage was not affected by light condition (see also Table 1), indicated by the absences of a significant main effect of condition [*F*(2,120) = 2.53; *P* = 0.084; *η*^2^_part._ = 0.04] or an interaction between age group and light condition [*F*(2,120) = 0.82; *P* = 0.445; *η*^2^_part._ = 0.01]. Only a main effect of age group emerged [*F*(1,60) = 45.51; *P* < 0.001; *η*^2^_part._ = 0.43], demonstrating that adolescents had significantly more N3 sleep than adults (see Fig. 4A). During the first night quarter, we not only observed the age effect [*F*(1,60) = 29.70; *P* < 0.001; *η*^2^_part._ = 0.33] but also an interaction between age group and light condition [*F*(2,120) = 4.36; *P* = 0.015; *η*^2^_part._ = 0.07], showing a reduction of early N3 sleep exclusively among adults when they read on a smartphone without a filter (No Filter versus Filter: *t*_32_ = −2.90; *P*_adj._ = 0.020; *d* = −0.26, No Filter versus Book: *t*_32_ = −2.38; *P*_adj._ = 0.036; *d* = −0.16). The percentage of N3 sleep during the first night quarter was, however, higher in adults who had stronger melatonin recovery rates after light exposure (rho_34_ = 0.41; *P* = 0.014, see Fig. 4A).

**Table 1:** Sleep architecture across the whole night, split by age group and light condition

	Adolescents (*N* = 30)			Adults (*N* = 32)		
	No Filter	Filter	Book	No Filter	Filter	Book
TIB (min)	480.77 (0.53)	480.18 (0.37)	480.13 (0.38)	480.08 (0.38)	479.91 (0.41)	479.86 (0.58)
TST (min)	466.18 (2.06)	465.30 (3.26)	466.38 (1.86)	449.95 (6.12)	452.70 (4.66)	463.08 (2.23)
EFF (%)	96.97 (0.40)	96.90 (0.68)	97.15 (0.36)	93.73 (1.27)	94.32 (0.94)	96.50 (0.41)
SOL N2 (min)	11.22 (1.51)	9.80 (1.08)	8.67 (0.85)	15.61 (2.24)	15.28 (2.18)	13.92 (1.68)
N1 (%)	5.96 (0.80)	5.47 (0.60)	5.87 (0.71)	13.38 (0.88)	13.06 (1.09)	12.57 (0.82)
N2 (%)	46.60 (1.33)	45.78 (1.18)	46.04 (1.27)	49.93 (1.14)	49.03 (1.17)	50.12 (1.10)
N3 (%)	28.84 (1.33)	29.73 (1.24)	28.15 (1.31)	17.67 (1.20)	17.89 (1.23)	17.46 (1.09)
REM (%)	18.60 (1.06)	19.02 (0.95)	19.94 (1.13)	19.03 (0.92)	20.01 (0.80)	19.86 (0.89)

Mean and standard error are presented. TIB, time in bed; TST, total sleep time; EFF, sleep efficiency; SOL N2, sleep onset latency until N2; REM, rapid eye movement sleep.

We further assessed the density of SOs during the first night quarter (i.e. SOs per minute of N3 sleep; see Fig. 4B). A significant interaction between ‘age group’ and ‘light condition’ [*F*(2,116) = 5.90; *P* = 0.004; *η*^2^_part._ = 0.09] indicated that adolescents had a significantly reduced SO density in the Filter than in the Book condition (*t*_27_ = −3.27; *P*_adj._ = 0.008; *d* = −0.30), but adults showed the opposite pattern with, by trend, an increased density in the Filter compared to the No Filter (*t*_27_ = 1.96; *P*_adj._ = 0.089; *d* = 0.40) and Book (*t*_27_ = 2.23; *P*_adj._ = 0.089; *d* = 0.50) conditions. Overall, adults showed a higher SO density than did adolescents [*F*(1,58) = 25.10; *P* < 0.001; *η*^2^_part._ = 0.30]. We did not observe any apparent effects of light condition on SWA, and interestingly, there was no clear age effect (see Supplementary Fig. 2).

**Figure 5:**
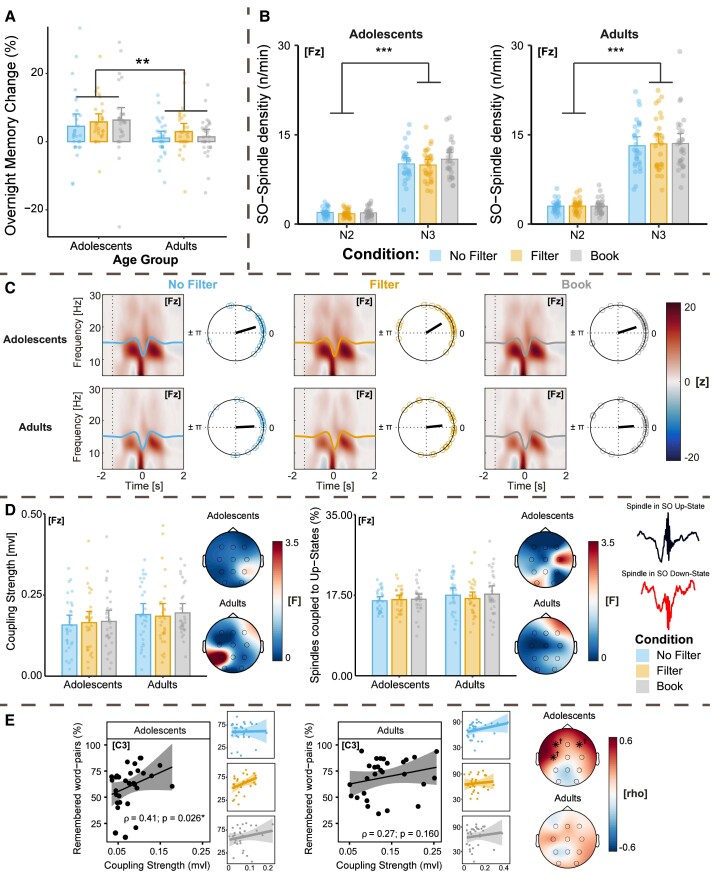
**Sleep-related memory consolidation**. (**A**) Overnight memory consolidation assessed by a mixed-design ANOVA. Adolescents benefited significantly more from the sleep retention interval than adults did [*F*(1,58) = 10.91; *P* = 0.002]. However, overnight memory consolidation was not affected by light condition [*F*(2,116) = 0.71; *P* = 0.495]. (**B**) Co-occurring SO–spindle events were significantly more frequent during N3 than N2 sleep in both adolescents and adults as assessed by two repeated measures ANOVAs [Adolescents: *F*(1,29) = 367.61; *P* < 0.001, Adults: *F*(1,29) = 201.13; *P* < 0.001]. (**C)** Time–frequency plots centred at SO troughs (down-states), showing an increase in spindle power (∼10–15 Hz) at the SO peaks (up-states), across all light conditions, together with a clear phase locking of spindles to the SO peak (zero on the circular plots). In adults, spindles arrived at the peak slightly more precisely, whereas the coupling was shifted more towards the descending phase of the peak (>0) in adolescents. (**D**) The coupling strength (mvl) and the percentage of spindles arriving at SO up-states did not differ significantly among light conditions (main effect of condition in the mixed-design ANOVA for coupling strength: *F*(2,116) = 0.44; *P* = 0.643 and for the percentage of coupled spindles: *F*(1.8,104.29) = 0.64; *P* = 0.512]. (**E**) Spearman rho correlations between coupling strength (mvl) and behavioural recall performance during the immediate recall of the word pair task for adolescents and adults. While the coupling strength was consistently positively correlated with learning performance, the results were only significant for adolescents and the relationship was weaker in adults. Significant (*P* < 0.050) electrodes are highlighted with an asterisk. Results that were not significant after correction for multiple testing are marked with a cross. ***P < 0.001. **P ≤ 0.010. *N*_Adolescents_ = 30 and *N*_Adults_ = 30. Bars display the mean and error bars the 95% confidence interval.

Finally, we assessed whether reading on a smartphone before bedtime had an impact on sleep-related memory consolidation. First, memory performance did not differ across the three lab visits [*F*(2,116) = 0.53; *P* = 0.589; *η*^2^_part._ = 0.01, see Supplementary Fig. 3], indicating no learning effect in the task. By trend, adults performed better than adolescents [*F*(1,58) = 3.68; *P* = 0.060; *η*^2^_part._ = 0.06], and memory performance in the morning was consistently higher than in the evening for both age groups [*F*(1,58 = 71.98; *P* < 0.001; *η*^2^_part._ = 0.55, see Supplementary Fig. 4A]. A significant interaction between recall timepoint (evening versus morning) and age group [*F*(1,58) = 10.34; *P* = 0.002; *η*^2^_part._ = 0.15] suggested that adolescents profited more from the sleep interval than did adults (see Fig. 5A for the overnight memory change analysis). We did not observe any significant effect of light condition [*F*(2,116) = 0.49; *P* = 0.615; *η*^2^_part._ = 0.01] and no interaction between light condition and recall timepoint [*F*(2,116) = 2.00; *P* = 0.140; *η*^2^_part._ = 0.03]. For the coupling between SOs and sleep spindles, we focussed on N3 sleep since the density of co-occurring SO–spindle events was significantly higher during N3 than during N2 (see Fig. 5B). We selected the electrode Fz for the coupling analyses, due to the frontal dominance of SOs. Co-occurring SO–spindle events were non-uniformly distributed in both age groups and clustered significantly at certain SO phase angles in all light conditions (all *Z* > 9.51 and *P* < 0.001).

On average, spindles were locked more precisely to the SO peak (up-state) in adults than in adolescents across all conditions, indicating an intra-individually robust SO–spindle coupling mechanism (see Fig. 5C). However, this effect was not affected by light condition, and the coupling strength (mvl), along with the percentage of spindles coupled to SO up-states, did not differ among conditions [mvl: *F*(2,116) = 0.44; *P* = 0.643; *η*^2^_part._ = 0.01, coupling percentage: *F*(1.8,104.29) = 0.64; *P* = 0.512; *η*^2^_part._ = 0.01, see Fig. 5D]. For a supplementary analysis of the data from the first night quarter, see Supplementary Fig. 4B–D, with highly similar results. We were also able to demonstrate that the coupling strength between sleep spindles and SOs was, indeed, positively correlated with individual learning performance especially in adolescents (see Fig. 5E), which conceptually replicates previous findings.^30,70^

## Discussion

In this study, we assessed whether evening short-wavelength light affects adolescents and young adults differently, not only with respect to acute sleepiness and melatonin suppression, but also in terms of effects on sleep architecture and physiology, especially sleep-related memory consolidation. While subjective sleepiness was not influenced by the light exposure, we observed a clear attenuation of melatonin secretion in both age groups. Interestingly, adolescents recovered more quickly, whereas adults experienced a suppression effect that persisted until bedtime. Consequently, only the adults exhibited slightly impaired sleep, as indicated by a reduction of N3 sleep during the first night quarter. However, adults with higher melatonin secretion rates and a quicker recovery from the melatonin attenuation were able to maintain higher percentages of N3 after the short-wavelength light exposure. Sleep-related memory consolidation remained unaffected in both age groups.

### Short-wavelength light effects on sleepiness and melatonin secretion

Our study did not replicate previous findings of reductions in subjective sleepiness due to evening short-wavelength light.^8-10,26,75^ Still, the fact that we observed a continuous increase in sleepiness throughout the evening and used the same questionnaire as in most prior studies^63^ indicates that the lack of light effects on sleepiness is unlikely due to methodological issues. In addition to variations in study designs and light sources—particularly in terms of melanopic intensities—our sleepiness ratings may not have been sensitive enough to detect subtle changes that participants were unaware of. This is especially so, since we observed clear physiological effects in melatonin attenuation, which were most pronounced after reading on the smartphone without a blue-light filter. Recent evidence suggests that light colour alone does not reduce melatonin secretion when melanopic intensity is held constant,^76^ yet our results do not contradict these findings, because our light conditions also varied significantly in melanopic intensity (see Fig. 1C). Overall, our results suggest that light-induced effects on circadian physiology, such as melatonin secretion, may occur without participant awareness. This aligns with recent literature indicating that alterations in melatonin levels may not necessarily correspond to subjective sensations like sleepiness or alertness, despite common misconceptions to the contrary.^42^

Our results demonstrate a higher melatonin secretion in adolescents than in young adults, a phenomenon that has been reported previously.^77,78^ Most likely, this is due to a gradual reduction in melatonin secretion across development, beginning in prepubertal children and continuing into late adulthood, probably due to reductions in pupil size and lens clarity.^79^ Alternatively, differences in previous light history could account for some of the results,^18^ although this would have required systematic differences between the two age groups which cannot be ruled out but does not seem likely. Consequently, we observed that a 50 min break between light exposure and bedtime was sufficient for the adolescents to recover from the light-induced melatonin suppression, but not for the adults. This contradicted our initial expectations, based on reports suggesting that adolescents are particularly sensitive to evening short-wavelength light.^5,80^ We believe that the longer-lasting melatonin attenuation in adults but not adolescents is due to the quicker melatonin recovery observed in adolescents, which is likely linked to their higher overall melatonin secretion rates.^81^ In addition, others failed to detect any significant age effects when comparing adolescents with young adults in terms of acute light-induced melatonin suppression,^82^ emphasizing the need for further research to comprehensively understand age-related changes in light sensitivity. Based on our findings, a possible explanation for this inconsistency could be that the acute melatonin suppression might indeed show small age-related differences, but that adolescents recover more quickly. This could have implications for sleep hygiene recommendations, as shorter breaks between the end of light exposure and bedtime might be sufficient for adolescents to avoid negative melatonin effects. Future studies should probe whether this can be further corroborated by empirical data and determine the minimum amount of ‘screen-free’ time necessary before bedtime to prevent sleep-disrupting effects.

### Impact on subsequent sleep architecture and sleep physiology

Despite sustained melatonin attenuation until bedtime in adults, longer-lasting effects on sleep were only marginal for them—and entirely absent for adolescents. While this is not the first time sleep-impairing effects could not be replicated,^41,83-85^ there remains a body of research advocating the existence of detrimental effects of LED screen usage before bedtime on sleep.^8,25,26,29,86^ Therefore, it is essential to gain a better understanding of the circumstances that may lead to sleep-disrupting effects of evening light exposure and to explore potential explanations for disparities in the existing literature.

First, subjective and objective sleep measures might not always align, and when sleep is assessed objectively, a further distinction must be made between actigraphy and PSG recordings, with the latter representing the gold standard. Even when focussing solely on PSG studies,^8,25,26,29,41,87^ several other factors may contribute to incomparability between studies and could account for conflicting findings. Above all, the duration of the light exposure and its timing relative to bedtime are pivotal factors influencing sustained light-induced effects. A minimum of 2 h of light exposure seems to be necessary to consistently observe significant effects on sleep architecture,^8,25,29^ with a single exception where 30 min of reading on a tablet immediately before bedtime sufficed to induce a delay and slight reduction in SWA.^26^ Given that our light exposure lasted only 90 min and concluded, on average, 50 min before bedtime, this might account for our limited sleep effects. Furthermore, screen size and melanopic irradiance also significantly modulate light-induced sleep effects,^9^ and only recently, a standardized metric of melanopic light intensity has been brought forward by the CIE S 026/E:2018 (i.e. the m-EDI). This metric has the potential to facilitate more straightforward comparisons between studies in the future.^88^

An additional, albeit speculative, explanation for the absence of sleep effects in our adolescent group could be related to differences in sleep pressure at bedtime that might have overruled harmful effects of the preceding light exposure. As evidenced by our sleepiness ratings (see Fig. 2), the adolescents were significantly more tired at bedtime than the adults, regardless of the light condition. This higher sleep pressure could be due to significant variations in sleep patterns that emerge from inconsistent bedtimes and sleep durations between school days and weekends.^87,89^ However, since the adolescents were asked to adhere to a consistent 8 h sleep schedule, we tried to avoid these variations, even if we cannot rule out that they nevertheless varied their bedtimes slightly more than the adults. Additionally, the 8 h sleep schedule was at the lower limit of the recommended sleep duration for adolescents, which is commonly prescribed as 8–10 h per night,^88,90^ but which perhaps is unrealistic to adhere to during normal school days.

### Implications for sleep-dependent memory consolidation

Finally, we tried to gain new insights into potential consequences of evening light exposure on more specific sleep-related processes, particularly declarative memory consolidation. While we could not detect any effects on behavioural or physiological indicators of sleep-dependent memory consolidation, this may be attributable to the general absence of light-induced effects on sleep physiology. Thus, it remains plausible that light-induced impairments in slow-wave sleep, as reported in prior studies,^25,26,29^ could indeed have led to disruptions in the interplay between SOs and sleep spindles (hallmark features of slow-wave sleep), constituting a crucial element of sleep-dependent memory consolidation.^30,32,91^ This is the first time that sleep-dependent memory consolidation was assessed in terms of potential short-wavelength light exposure. Therefore, it is not yet justified to make strong claims regarding the effect (or lack of one) of light exposure on sleep-dependent memory consolidation. Previous literature has focussed on only the positive effects of prelearning light exposure on subsequent memory performance,^92,93^ but these effects might be reversed in nighttime light exposure and subsequent sleep. Future studies with longer exposure durations (>2 h) closer to bedtime (<30 min) and potentially higher melanopic illuminances would be required to unravel whether an impairment of sleep-dependent memory consolidation is a justified concern.

### Limitations

Even though we tried to mimic a naturalistic and ecologically valid study design, there remains a list of limitations that might have influenced our results. First, we had a plethora of inclusion criteria that led to a rather selective sample. For instance, we included only male participants, due to reported sex effects on light sensitivity, sleep physiology and circadian rhythmicity,^47,94-96^ which we tried to avoid, as controlling for these effects would have demanded an even larger sample size that would not have been feasible given the study design. Due to the high specificity of our sample, our results need to be generalized with caution. Second, our participants were allowed to read only preselected stories during the light exposure, which probably does not reflect real-life smartphone behaviour. However, this restriction was necessary to assure a standardized experimental condition that allowed for an equivalent control condition without screen exposure. As most users probably spend their screen time primarily with more engaging activities, like social media, gaming or watching short video clips, future studies should try to disentangle the effects of light exposure from additional arousing effects of the performed activities. While recent studies suggest that the use of social media or induced suspense from cliffhangers in TV series only minimally affect subsequent sleep,^91,97^ research directly comparing the exclusive effects of short-wavelength light with additional activities on light-emitting devices is scarce. Lastly, our 50 min break before bedtime does not entirely reflect typical smartphone use, since most adolescents and adults use their phone until 30 min before bedtime or even immediately preceding, while lying in bed.^92^ Unfortunately, a shorter break before bedtime was not feasible with our design, since our study included additional tests and questionnaires after the light exposure, as well as a final bathroom break and preparations for the PSG recording.

## Conclusion

Nighttime smartphone reading does not appear to interfere with whole-night sleep and sleep-dependent memory consolidation in adolescents and young adults if the smartphone is put away 1 h before bedtime. Although smartphone usage, especially without a blue-light filter, exerts pronounced suppressive effects on melatonin, adolescents demonstrate a more rapid recovery from the melatonin suppression than young adults do. In conclusion, it is advisable for adolescents and young adults to avoid exposure to LED screens in the last hour before bedtime, which is in line with general sleep hygiene recommendations.^93^ Future research is warranted to ascertain whether this 1-h window is indeed the minimum required before bedtime or whether shorter breaks might be sufficient to mitigate sleep disturbances.

## Supplementary Material

fcae173_Supplementary_Data

## Data Availability

The data and scripts for analyses underlying this article will be shared upon request to the corresponding authors.
