# Supplementary material for: Effects of evening smartphone use on sleep and declarative memory consolidation in male adolescents and young adults
Source: Brain Commun. 2024 May 17;6(3):fcae173. doi: 10.1093/braincomms/fcae173 (PMC11154150; doi:10.1093/braincomms/fcae173)
Supplement: fcae173_Supplementary_Data [file fcae173_supplementary_data.docx]

**Supplementary File – Effects of evening smartphone use on sleep and declarative memory consolidation in male adolescents and young adults**

**Supplementary Table 1.** Entrance screening questionnaire results.

| **Questionnaire** | **Age Group** | **Mean (SD)** | **Cut-Off** |
| --- | --- | --- | --- |
| Pittsburgh Sleep Quality Index (PSQI)^1^ | Adolescents | 3.58 (2.55) | ≥ 10 |
|  | Adults | 3.43 (1.97) |  |
| State Trait Anxiety Inventory: Trait (STAI-T)^2^ | Adolescents | 34.18 (8.45) | ≥ 45 |
|  | Adults | 33.86 (8.59) |  |
| Social Interaction Anxiety Scale (SIAS)^3^ | Adolescents | 20.42 (10.20) | ≥ 30 |
|  | Adults | 24.03 (16.12) |  |
| Beck-Depression-Inventory (BDI-II)^4^ | Adolescents | 5.30 (5.06) | ≥ 18 |
|  | Adults | 4.17 (4.54) |  |
| Perceived Stress Scale (PSS)^5^ | Adolescents | 12.94 (5.77) | ≥ 27 |
|  | Adults | 10.77 (5.75) |  |
| Smartphone Addiction Scale (SAS)^6^ | Adolescents | 24.12 (8.76) | ≥ 31 |
|  | Adults | 20.51 (5.90) |  |
| Morning-Eveningness Questionnaire (D-MEQ)^7^ | Adolescents | 44.52 (7.59) | ≤ 30 or ≥ 70 |
|  | Adults | 53.74 (8.43) |  |

Note. Cut-Off values refer to recommended critical values provided in the questionnaire’s manual if available. For the State Trait Anxiety Inventory (STAI-T), cut-off values recommended by Stojanović et al.^8^ were used as no official recommendations were provided in the test manual.

**Supplementary Table 2.** Books that were used for the reading session (light exposure).

| **Age Group** | **Title** | **Author** | **Published** |
| --- | --- | --- | --- |
| Adolescents | 555 Populäre Irrtümer: Warum Angela Merkel eigentlich ein Wessi ist, man Eier nicht abschrecken muss und Erdnüsse keine Nüsse sind | Golluch, N. | 2014 |
| Adolescents | 13 gegen das Sommerloch: 13 Autoren – 13 Geschichten – 13 x Lesespaß | Wölk,  J. | 2015 |
| Adults | Eragon: Das Vermächtnis der Drachenreiter | Paolini, C. | 2003 |
| Adults | Herr der Diebe | Funke,  C. | 2000 |
| Adults & Adolescents | Die Känguru Chroniken: Ansichten eines vorlauten Beuteltiers | Kling, M.U. | 2009 |

Note. Each book was read for 25min during the 90min light exposure in randomized order.

**Supplementary Table 3.** Time elapsed between the end of the light exposure and bedtime (i.e., lights out).

| **Age Group** | **Light Condition** | **Mean (SD)** | **Min** | **Max** |
| --- | --- | --- | --- | --- |
| Adolescents | No Filter | 46min (5.3) | 38min | 67min |
| Adults | No Filter | 53min (6.7) | 41min | 69min |
| Adolescents | Filter | 47min (9.6) | 19min | 74min |
| Adults | Filter | 52min (7.9) | 20min | 70min |
| Adolescents | Book | 46min (6.4) | 38min | 62min |
| Adults | Book | 51min (6.5) | 41min | 67min |

Note. No significant differences between the gap to bedtime across conditions (*p* = .632; *η²_part_* = .01) and no interaction between age group and condition (*p* = .730; *η²_part_* = .01). However, on average, adolescents had a significantly shorter gap to bedtime than adults (*p* < .001; *η²_part_* = .21).

**Supplementary Table 4.** Subjective ratings regarding room-light brightness (1 = not bright at all, 10 = extremely bright) and exhaustion during the reading task (1 = not exhausting, 5 = very exhausting). Mean and standard error of the mean are presented, p-values are obtained from non-parametrical ANOVA type statistics.

|  | **No Filter** | **Filter** | **Book** | **p-value** |
| --- | --- | --- | --- | --- |
| Brightness | 3.78 (0.17) | 3.96 (0.16) | 3.73 (0.21) | .256 (n.s.) |
| Exhaustion | 2.15 (0.12) | 1.99 (0.10) | 1.99 (0.11) | .750 (n.s.) |

Note. Ratings were given before going to bed on each experimental night.

**Supplementary Table 5.** Light source characteristics measured by the JETI spectrometer.

| **Age Group** | **Condition** | **m-EDI (lux)** | **Illuminance (lux)** | **CCT (K)** |
| --- | --- | --- | --- | --- |
| Adolescents (A50) | No Filter | 268.24 | 294.35 | 6317 |
| Adults (A51) | No Filter | 279.17 | 299.31 | 6072 |
| Adolescents (A50) | Filter | 80.27 | 205.54 | 2447 |
| Adults (A51) | Filter | 93.65 | 216.93 | 2527 |
| Adolescents / Adults | Book | 1.34 | 3.31 | 2470 |

Note. m-EDI = melanopic equivalent daylight illuminance. CCT = correlated color temperature. A50 and A51 = Smartphone models that were used for the light exposure: Samsung Galaxy A50 and Samsung Galaxy A51. Light measurements in the Book condition were equal for both age groups since the only active light source was the background room lighting.

**Supplementary Table 6.** Technical comparison between the Salimetrics melatonin assay, which was used for the adult sample and the Novolytix assay that was used for the adolescents.

|  | **SALIMETRICS** | **NOVOLYTIX** |
| --- | --- | --- |
| Analytical Sensitivity | 1.37 pg/mL | <0.5 pg/mL |
| Functional Sensitivity | 2.25 pg/mL | 1.3 pg/mL |
| Intra-assay Precision (Mean CV %) | 5.4 | 7.9 |
| Inter-assay Precision (Mean CV %) | 8.9 | 14.6 |
| Assay Range | 0.78 – 50 pg/mL | 0.5 – 50 pg/mL |


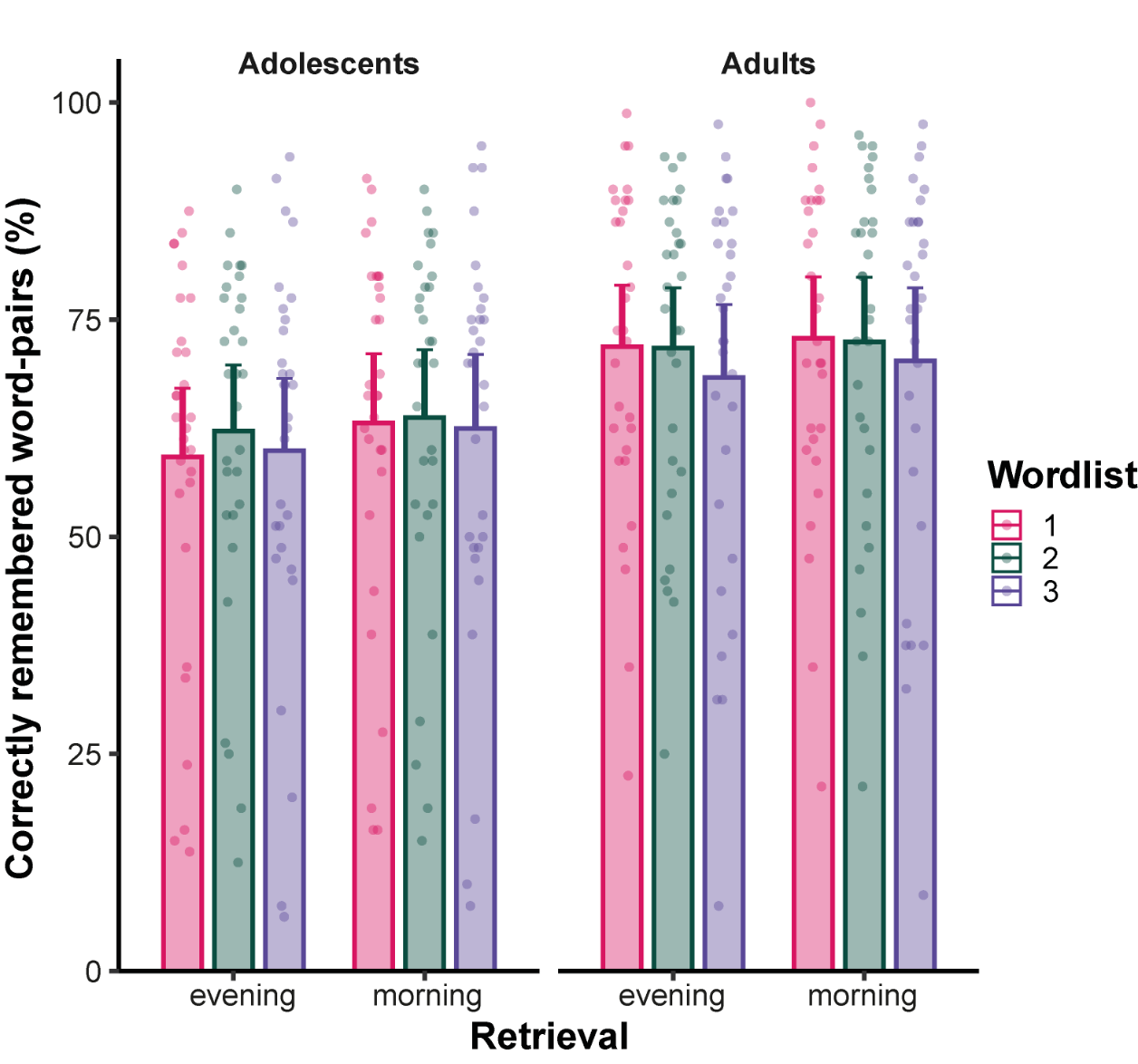


**Supplementary Figure 1.** The different wordlists used on each lab-visit do not significantly impact recall performance (main effect of wordlist: F_2, 116_ = 0.82; p = 0.443, interaction between age group and wordlist: F_2, 116_ = 0.39; p = 0.679), assessed with a mixed-design analysis of variance (ANOVA). N_Adolescents_ = 30 and N_Adults_ = 30. Bars display the mean and error bars, the 95% confidence interval. Individual data points represent single subject values.


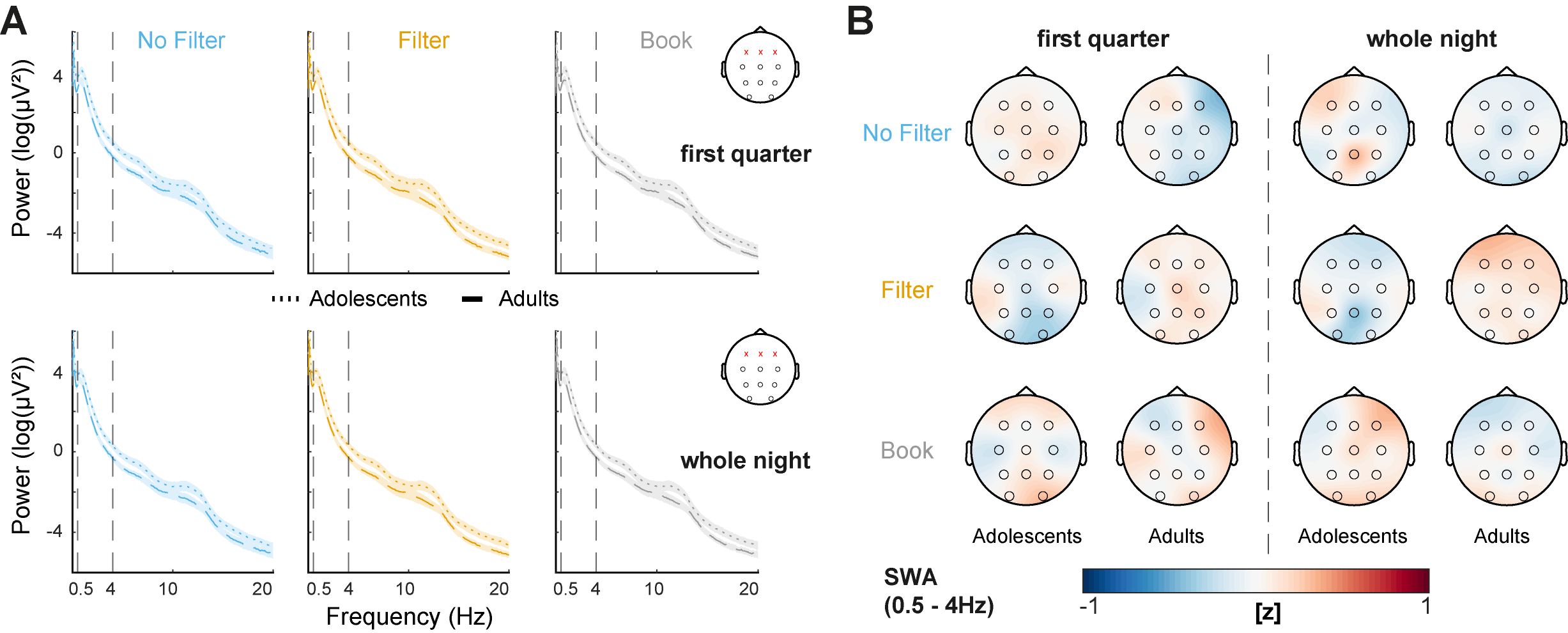


**Supplementary Figure 2.** A: Average semi-log power spectra during N3 sleep over frontal electrodes (F3, Fz, F4) for the first night quarter (top row) and the whole night (bottom row), depicting very similar results. B: Topographical distribution of slow-wave activity in z-normalized units relative to the subjects’ average value across the light conditions, per electrode. Slow-wave activity did not deviate significantly from the subject’s average activity in any of the light conditions (all z-values < 1).


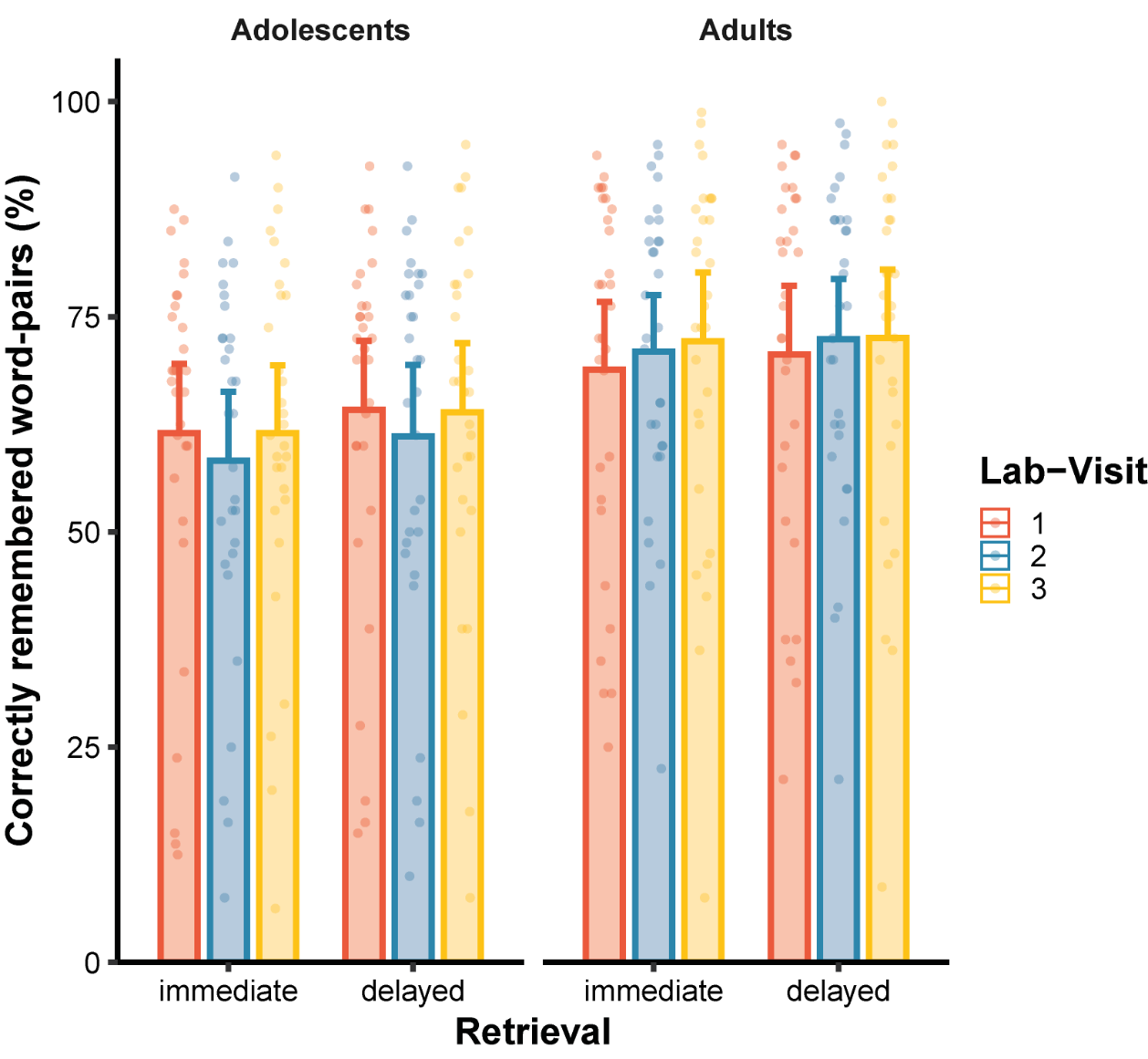


**Supplementary Figure 3.** Recall performance does not differ significantly between the lab-visits (main effect: F_2, 116_ = 0.53; p = 0.589, interaction between age group and lab-visit: F_2, 116_ = 0.99; p = 0.376) as assessed with a mixed-design analysis of variance (ANOVA). N_Adolescents_ = 30 and N_Adults_ = 30. Bars display the mean and error bars, the 95% confidence interval. Individual data points represent single subject values.


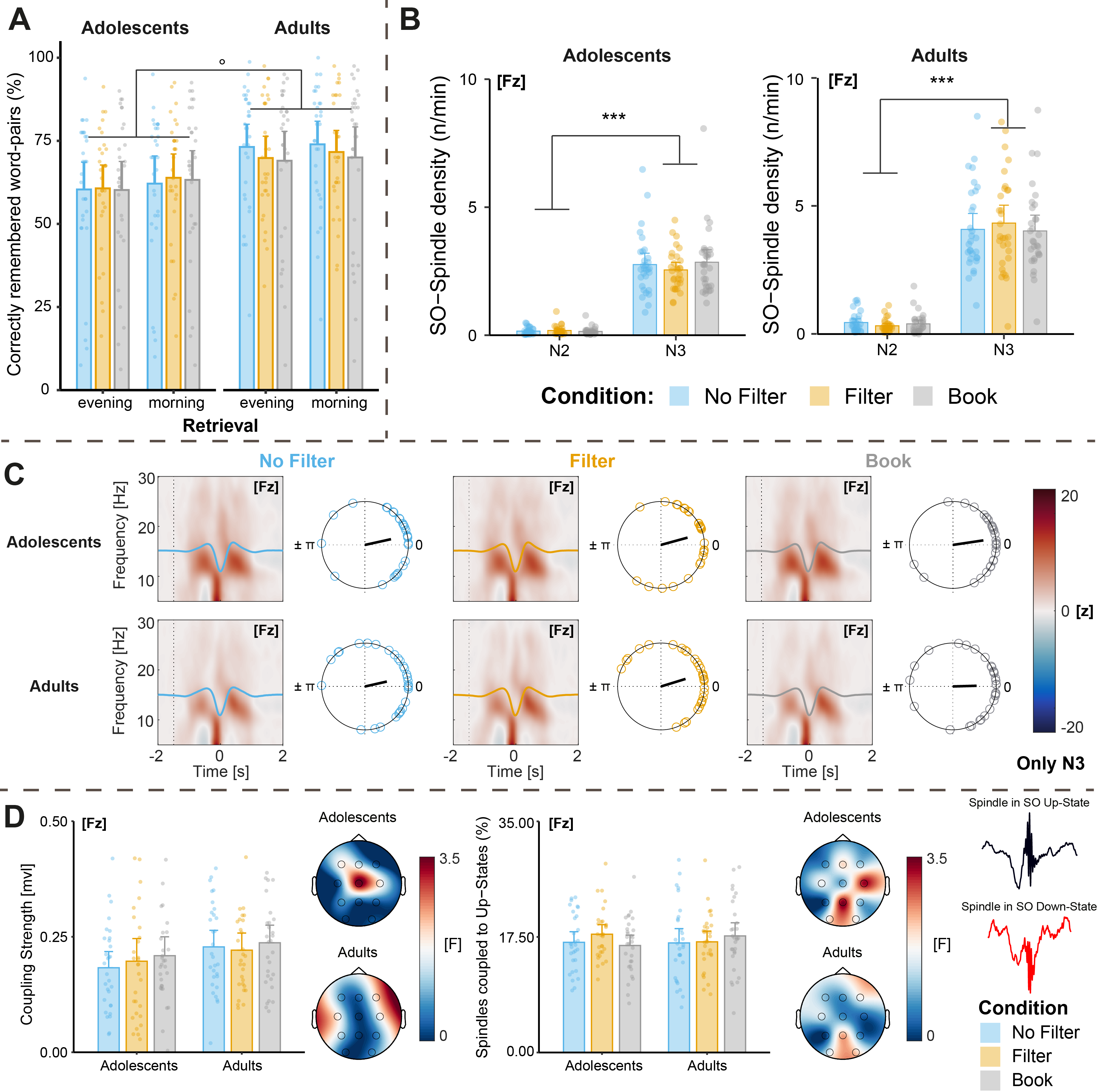


**Supplementary Figure 4.** A: Raw performance scores in the declarative learning task for the evening (immediate) and morning (delayed) recall, indicating better overall task performance in the adults as assessed with a mixed-design analysis of variance (ANOVA, main effect of age group: F_1, 58_ = 3.68; p = 0.060). B: Co-occurring SO-Spindle events during the first night quarter were significantly more frequent in N3 than N2 sleep for adolescents and adults as assessed by two repeated measures ANOVAs (Adolescents: F_1, 29_ = 178.50; p < 0.001, Adults: F_1, 29_ = 173.32; p < 0.001). C: Time-frequency plots centered at slow oscillation (SO) troughs during the first night quarter, showing an increase in spindle power (~ 10 – 15Hz) at the SO peaks (up-states) across all light conditions, together with a clear phase locking of spindles to the SO peak (0 on the circular plots). D: The coupling strength (mean vector length, mvl) and percentage of spindles arriving at SO up-states did not differ significantly among light conditions during the first night quarter (main effect of condition in the mixed-design ANOVA for coupling strength: F_2, 116_ = 1.09; p = 0.341 and for the percentage of coupled spindles: F_2, 116_ = 0.58; p = 0.564). ***: p < 0.001, °: p ≤ 0.100, N_Adolescents_ = 30 and N_Adults_ = 30. Bars display the mean and error bars, the 95% confidence interval. Individual data points represent single subject values in the according variables.

Supplementary References

1. Buysse DJ, Reynolds CF, Monk TH, Berman SR, Kupfer DJ. The Pittsburgh Sleep Quality Index: a new instrument for psychiatric practice and research. *Psychiatry Res*. 1989;28(2):193-213. doi:10.1016/0165-1781(89)90047-4.

2. Spielberger CD, Gorsuch RL, Lushene RE. *State-Trait Anxiety Manual*. Menlo Park, CA: Mind Garden, Inc; 1970.

3. Mattick RP, Clarke JC. Development and validation of measures of social phobia scrutiny fear and social interaction anxiety. *Behav Res Ther*. 1998;36(4):455-470. doi:10.1016/S0005-7967(97)10031-6.

4. Hautzinger M, Keller F, Kühner C. *Beck Depressions-Inventar (BDI-II): Revision*. Frankfurt am Main: Harcourt Test Services; 2006.

5. Cohen S, Kamarck T, Mermelstein R. A global measure of perceived stress. *Journal of Health and Social Behavior*. 1983;24(4):385-396.

6. Kwon M, Kim D-J, Cho H, Yang S. The smartphone addiction scale: development and validation of a short version for adolescents. *PLoS One*. 2013;8(12):e83558. doi:10.1371/journal.pone.0083558.

7. Griefahn B, Künemund C, Bröde P, Mehnert P. Zur Validitat der deutschen Ubersetzung des Morningness-Eveningness-Questionnaires von Horne und Ostberg. The Validity of a German Version of the Morningness-Eveningness-Questionnaire Developed by Horne and Ostberg. *Somnologie*. 2001;5(2):71-80. doi:10.1046/j.1439-054X.2001.01149.x.

8. Stojanović N, Ranđelović P, Nikolić G, et al. Reliability and validity of the Spielberger’s State-Trait Anxiety Inventory (STAI) in Serbian university student and psychiatric non-psychotic outpatient populations. *Acta fac medic Naissensis*. 2020;37(2):149-159. doi:10.5937/afmnai37-25011.
